# Interfacial chemistry of biochar-modified cementitious materials: mechanisms of pore structure refinement, chloride immobilization, and carbon sequestration

**DOI:** 10.1039/d6ra00419a

**Published:** 2026-04-30

**Authors:** Lewei Wang, Liwei Song

**Affiliations:** a Henan Architecture Design & Research Institute Co. Ltd Zhengzhou Henan 450014 China; b Zhongyuan Institute of Science and Technology Xuchang Henan 461000 China slwill1@163.com

## Abstract

Cement manufacture remains a major source of anthropogenic carbon dioxide, motivating additives that can reduce net climate impact while improving service performance. This review discusses mechanistic evidence on how pyrolyzed biomass solids interact with hydrating Portland cement systems and how those interactions propagate from the particle scale to bulk durability. Three coupled pathways are critically discussed. First, surface oxygen containing groups and high specific area promote calcium enrichment and provide templates for calcium silicate hydrate (CSH) precipitation, shifting hydration kinetics and densifying the interfacial transition zone (ITZ). In parallel, water stored within the hierarchical porosity of the particles can be released during self-desiccation, supporting continued hydration and limiting early age cracking through internal curing. Second, resistance to salt exposure is mainly improved indirectly: matrix densification reduces connectivity and increases tortuosity of transport pathways, lowering apparent diffusion and migration metrics. Possible direct anion uptake by the carbonaceous phase is evaluated as an open question because high pore solution alkalinity tends to render surfaces negatively charged, and reported outcomes vary with feedstock, pyrolysis temperature, and post treatments. Third, climate benefits arise from stable biogenic carbon storage and, in some systems, accelerated carbonation that increases calcium carbonate formation. The review highlights a key durability trade-off for reinforced concrete, where faster carbonation may reduce alkalinity and promote depassivation of steel. Finally, priorities are proposed for rational mix design, including property standardization, advanced nanoscale characterization, and long duration field validation to reconcile laboratory trends with structural risk and life cycle outcomes in practice.

## Abstract 2

The role of biochar in cementitious materials is reviewed through a conceptual pathway.

## Graphical abstract

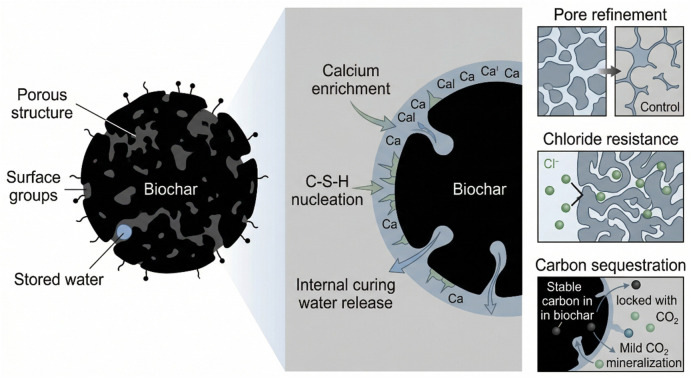

## 1. Introduction

The construction industry, with cement production as its cornerstone, is a significant contributor to global anthropogenic greenhouse gas emissions, accounting for approximately 8% of the world's total CO_2_ output.^1^ The calcination of limestone (CaCO_3_ → CaO + CO_2_) and the combustion of fossil fuels to achieve the high temperatures required for clinkerization are the primary sources of these emissions.^2^ As global urbanization continues to accelerate, the demand for concrete, the most consumed man-made material on Earth, is projected to rise, exacerbating its environmental impact.^3^ This unsustainable trajectory has created an urgent imperative within the scientific and engineering communities to develop innovative strategies that mitigate the carbon footprint of concrete.^4^ These strategies range from the development of alternative low-carbon binders and supplementary cementitious materials (SCMs) to the integration of carbon capture and utilization technologies within the material itself.^5^

Within this context, biochar has emerged as a uniquely promising material. Biochar is a stable, carbon-rich solid produced through the thermochemical conversion of biomass in an oxygen-limited environment, a process known as pyrolysis.^6^ Historically recognized for its agricultural benefits in soil amendment, its application in cementitious materials is a relatively recent but rapidly growing field of research.^7^ The allure of biochar lies in its multifaceted potential.^8^ Firstly, the pyrolysis process transforms labile carbon from biomass into a highly recalcitrant aromatic structure, effectively locking it away for centuries to millennia. Incorporating this stable carbon into the long service life of concrete structures presents a direct and scalable pathway for carbon sequestration.^9^ This positions biochar not merely as a ‘less polluting’ additive but as a potentially ‘carbon-negative’ component, actively removing CO_2_ from the atmosphere over its life cycle.^10^

Beyond its carbon sequestration credentials, biochar imparts significant modifications to the properties of fresh and hardened cementitious composites. Its porous structure, high specific surface area, and reactive surface functional groups fundamentally alter the hydration kinetics and microstructural development of the cement paste.^11^ These modifications can lead to tangible improvements in mechanical strength, a reduction in autogenous shrinkage, and enhanced durability against aggressive environmental agents. The interfacial chemistry between the biochar particles and the evolving matrix of cement hydration products is central to these performance enhancements.^12^ Understanding these complex interactions at a fundamental level is paramount for optimizing the design and application of biochar-modified concrete.^13^

**Fig. 1:**
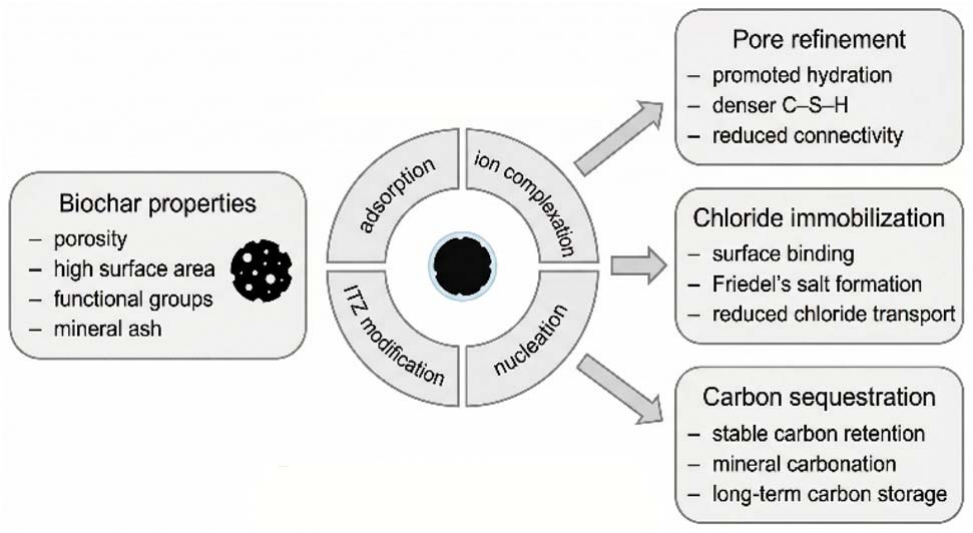
Graphical overview of the interfacial-chemistry-driven roles of biochar in cementitious materials.

Fig. 1 provides a graphical roadmap of the review, highlighting how biochar physicochemical features translate into interface-governed mechanisms and, ultimately, three performance pillars: pore structure refinement, chloride immobilization, and carbon sequestration. This framework is used throughout the manuscript to connect molecular-scale interfacial chemistry with durability- and sustainability-relevant outcomes.^14^ While several recent and comprehensive reviews have thoroughly documented the macroscopic mechanical properties, general sustainability metrics, and accelerated CO_2_ curing of biochar–cement composites, there remains a critical gap in linking these bulk behaviors to fundamental molecular interactions.^15–18^ This review paper aims to bridge that gap by providing a critical examination of the state-of-the-art understanding of the interfacial chemistry in biochar–cementitious systems. By isolating nanoscale phenomena—such as surface functional group chelation, localized supersaturation, and pore-solution thermodynamics—this work uniquely maps how chemical interactions at the biochar–cement interface dictate pore structure refinement, chloride immobilization, and the carbonation–corrosion dilemma.^19^ The first section explores the molecular-level interactions that govern cement hydration and lead to the refinement of the material's pore structure. The second section delves into the mechanisms of chloride immobilization, a crucial factor for the durability of reinforced concrete in marine or de-icing salt environments, analyzing the direct and indirect roles of biochar. The third section critically evaluates the dual mechanisms of carbon sequestration and confronts the scholarly debate surrounding its long-term stability and potential consequences, particularly the risk of reinforcement corrosion. By synthesizing experimental evidence, discussing conflicting findings, and highlighting current controversies, this review seeks to advance the chemical understanding necessary for harnessing the full potential of biochar as a sustainable construction material.^20^

Several recent reviews have summarized the broad application, performance, and carbon-abatement roles of biochar in cementitious materials, but comparatively fewer studies have organized the field around the interfacial chemistry that couples hydration, pore refinement, chloride partitioning, and carbonation-related durability. This revised review therefore emphasizes how biochar surface functionality, ash chemistry, pore architecture, and interaction with binder phase assemblage jointly control the three outcome domains discussed throughout the manuscript.^21–23^

## 2. Interfacial chemistry and hydration mechanisms

The performance of biochar-modified cementitious materials is fundamentally dictated by the complex interplay at the interface between the biochar particles and the surrounding cement paste.^23^ These interactions are not merely physical; they involve a suite of chemical reactions and surface phenomena that begin the moment biochar is mixed with cement and water, and continue throughout the hydration and hardening process.^24,25^ The unique physicochemical properties of biochar, which are tunable through the choice of feedstock and pyrolysis conditions, are central to these interfacial mechanisms.^26^

The surface of biochar is typically adorned with a variety of oxygen-containing functional groups, including carboxyl (–COOH), hydroxyl (–OH), and phenolic groups.^27^ The abundance and type of these functional groups are heavily dependent on the pyrolysis temperature, with lower temperatures generally yielding a higher density of such groups.^28^ These functional groups are critical as they provide chemically active sites for interaction with the ions present in the cement pore solution. Immediately upon mixing, cement grains, primarily tricalcium silicate (C_3_S) and dicalcium silicate (C_2_S), begin to dissolve, releasing calcium (Ca^2+^), silicate (Si(OH)_4_), and hydroxyl (OH^−^) ions into the aqueous phase, rapidly increasing the solution's pH.^29,30^

**Fig. 2:**
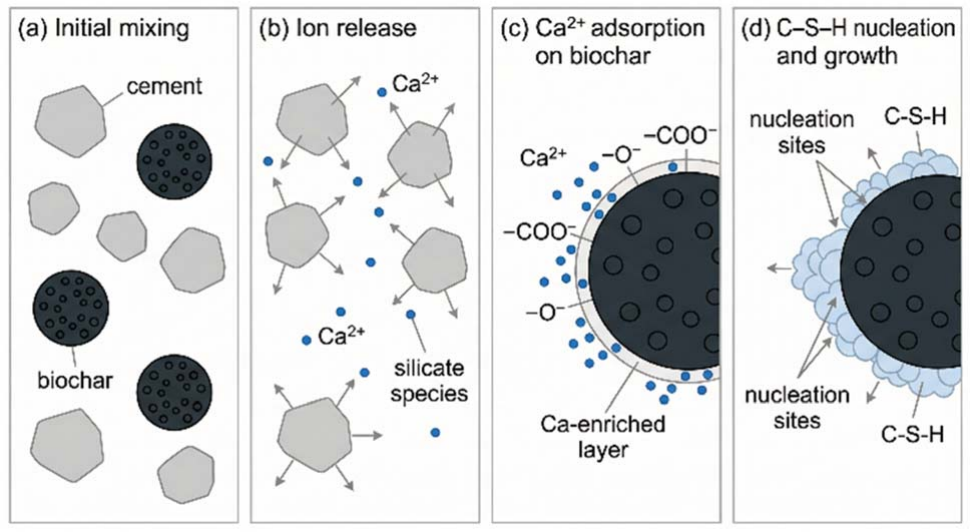
Simplified schematic of the nucleation role of biochar in cement paste. (a) Initial mixing of cement grains and biochar particles in water. (b) Release of calcium and silicate species from cement grains. (c) Accumulation of Ca^2+^ on oxygen-containing groups at the biochar surface. (d) Nucleation and growth of C–S–H near the biochar surface, promoting local hydration.

The negatively charged carboxylate groups (–COO^−^) and deprotonated hydroxyl groups (–O^−^) on the biochar surface act as potent chelating sites for the abundant divalent Ca^2+^ ions.^31^ This electrostatic attraction leads to the adsorption of Ca^2+^ ions onto the biochar surface, creating a calcium-rich layer.^32^ This localized supersaturation of calcium ions is hypothesized to be a primary driver of the “nucleation effect,” a phenomenon widely reported in the literature.^33,34^ As shown schematically in Fig. 2, the calcium-enriched biochar surface provides energetically favorable sites for the precipitation of primary hydration products, most notably calcium–silicate–hydrate (C–S–H) gel, which is the principal binding phase responsible for the strength of concrete. By lowering the activation energy barrier for nucleation, biochar particles act as templates, promoting the formation of a higher density of smaller, more uniformly distributed C–S–H clusters throughout the matrix, rather than allowing them to grow only on the surfaces of the original cement grains.^35,36^

At the same time, a critical reading of the experimental literature suggests that “nucleation” is often entangled with at least two other mechanisms that can produce superficially similar calorimetric signatures: ionic effects from ash or dopants, and internal curing *via* water uptake and delayed release. For example, Chen *et al.*^37^ engineered algal biochar–metal nanocomposite particles and used isothermal calorimetry to show that a 3 wt% algal biochar–Zn formulation delayed the main heat evolution peak from 8.3 to 10.0 h, while 3 wt% algal biochar–Ca produced a slight acceleration from 8.3 to 8.2 h; importantly, both variants increased 28 day compressive strength by 22.6% and 17.0%, respectively. This is instructive for interfacial-chemistry framing because it implies that the same carbonaceous scaffold can support strength gains consistent with heterogeneous nucleation and microstructural refinement, yet the early-age kinetics can flip direction depending on whether a metal-bearing phase promotes passivating surface layers (for Zn) or provides additional Ca-bearing nucleation chemistry (for Ca). In other words, “biochar” is not a single chemical perturbation: it can be a carrier of distinct inorganic interfacial chemistries that must be separated experimentally if mechanistic claims are to remain robust.

This accelerated (or altered) formation of hydration products has also been verified using techniques that go beyond calorimetry alone. In biochar-augmented 3D printable cementitious mixtures, Wang *et al.*^38^ reported that incorporating 2 wt% biochar promoted hydration and increased polymerization of hydration products during the initial resting period, supported by *in situ* mineralogical probes, while simultaneously reducing the carbon footprint of the printable concrete by 8.3%. Mechanistically, this kind of result strengthens the interfacial narrative because it supports a coupled pathway: biochar acts as a hydrate growth substrate (an interfacial nucleation template)^39^ and as a water reservoir that modifies local water availability, which can accelerate dissolution and precipitation even when the nominal water-to-binder ratio is unchanged.^40,41^

However, the effect of biochar on hydration is not without controversy. Some studies have reported a delay in the initial hydration peak, attributing this to the adsorption of alkali ions (Na^+^, K^+^) from the pore solution onto the biochar.^42^ This adsorption can temporarily reduce the alkalinity and ionic strength of the pore solution, slightly slowing the initial dissolution of cement clinker phases.^43^ Furthermore, the high specific surface area of some biochars can adsorb chemical admixtures like superplasticizers, reducing their effective concentration and potentially altering the hydration kinetics.^11^ From a critical standpoint, these reported effects also highlight a methodological risk: hydration changes inferred from heat flow can reflect dispersion state and admixture availability as much as intrinsic interfacial reaction barriers.^44^ Because polycarboxylate ether-type superplasticizers interact through adsorption equilibria and can measurably delay hydration when adsorption characteristics change, admixture depletion by a high-surface-area additive can become a confounding variable unless explicitly controlled (for example, by measuring residual admixture concentration or adsorption isotherms).^45^ Thus, mechanistic attribution to biochar functional groups alone is weakest when admixture compatibility and pore-solution chemistry are not independently characterized.

These observations highlight that hydration control in biochar-modified binders is not governed by a single mechanism. Reported shifts in calorimetry, setting, and later-age strength reflect the competition among heterogeneous nucleation, internal curing, ash-driven ionic effects, admixture adsorption, and dosage-dependent dilution. Accordingly, the broader literature now suggests that the direction and magnitude of hydration changes depend strongly on biochar chemistry, associated inorganic phases, particle size distribution, and replacement level. This mechanistic heterogeneity helps explain why chloride resistance and pore refinement may improve at optimized low dosages yet deteriorate once excessive porous replacement begins to increase connected void space. Table 1 synthesizes these hydration-related studies by aligning feedstock, processing, measurement basis, and mechanistic interpretation rather than treating all kinetic shifts as evidence of a single universal nucleation pathway.

**Table 1:** Varied effects of biochar from different sources and pyrolysis conditions on cement hydration^a^

Biochar feedstock	Pyrolysis temp. (°C)	Dosage (wt% cement)	Observed effect on hydration	Proposed mechanism	Reference
Wood waste	500	1–3	Accelerated main hydration peak	Nucleation effect due to surface functional groups	46
Rice husk	700	2	Increased cumulative heat release after 24 h	Pozzolanic reaction of silica-rich ash and nucleation	47
Sewage sludge	600	5	Delayed initial heat flow peak but higher total heat	Adsorption of alkali ions; later-age pozzolanic activity	48
Corn stover	450	1	Accelerated setting time and early strength gain	High surface area and water absorption providing nucleation sites	25
Coconut shell	800	0.5–2	Initial delay followed by acceleration	Adsorption of admixtures; high surface area for nucleation	44

a ‘Accelerated/delayed’ refers to the specific metric reported in the cited study. Unless otherwise stated, main hydration peak time (*t*_peak_) and cumulative heat (*Q*_*t*_) refer to isothermal conduction calorimetry reporting (heat-flow peak time; integrated heat at fixed ages) consistent with standard calorimetry practice (*e.g.*, ASTM C1702). ‘Setting time’ refers to Vicat needle measurements (initial/final setting as reported; *e.g.*, ASTM C191 or EN 196-3). Where the source provides comparable ages, cumulative heat is reported at 24 h/72 h/7 d; otherwise the qualitative trend is retained with the study-specific age/test name stated.

High-replacement carbon-storage strategies illustrate an additional boundary condition for the interfacial-hydration framework developed in this review. When biochar acts as a major cement replacement rather than a minor interfacial modifier, dilution, water-demand penalties, and reduced hydrate volume can outweigh local nucleation benefits. This balance is critical because maximizing carbon storage is not automatically compatible with the microstructural densification required for low permeability and chloride resistance; the outcome depends on whether particle engineering and binder design can preserve hydrate connectivity while accommodating the added porous phase.

**Fig. 3:**
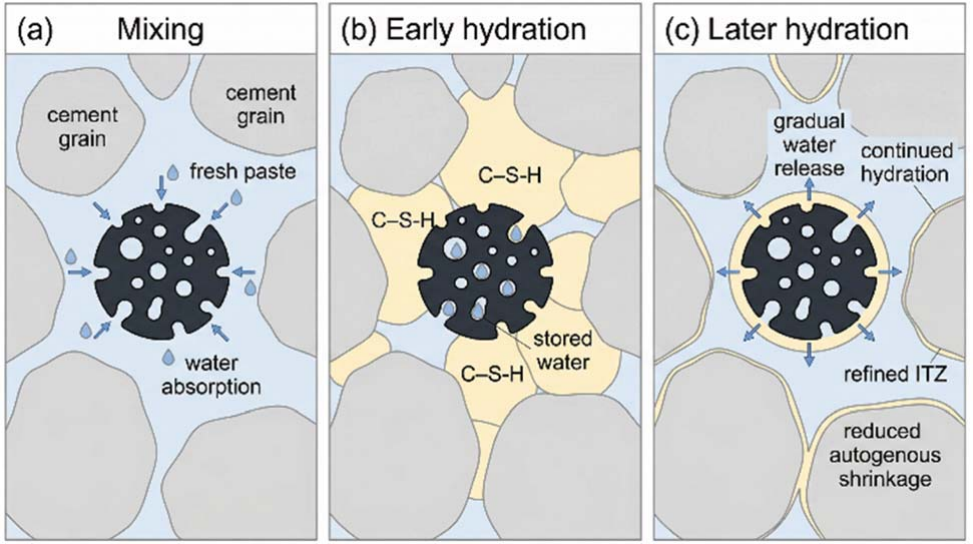
Simplified schematic showing how porous biochar provides internal curing during early and later hydration in a cementitious matrix.

Another crucial chemical and physical mechanism at the interface is “internal curing.” Biochar possesses a hierarchical porous structure, with micropores, mesopores, and macropores. During mixing, these pores absorb a significant amount of water. As hydration proceeds and consumes free water from the capillary pores, the relative humidity within the paste drops. This drop creates a potential gradient that draws the stored water out of the biochar pores and back into the matrix, as depicted in Fig. 3. This process of internal curing is particularly effective at mitigating autogenous shrinkage, which is caused by the chemical volume reduction (Le Chatelier contraction) during hydration and is a major cause of early-age cracking in low water-to-cement ratio concretes.^46^ In a quantitative example at the ultra-high-performance concrete (UHPC) scale, Du *et al.*^47^ reported that substituting cement with 1% pre-saturated waste-derived biochar increased mechanical strengths by up to 20% and toughness by up to 25%, while reducing autogenous shrinkage by up to 15%, explicitly attributing the shrinkage mitigation to the internal curing water released from biochar pores during early hydration.^48^ At the cement paste scale, Mo *et al.*^49^ similarly showed that incorporating biochar maintained a higher internal relative humidity and reduced autogenous shrinkage by 16.3% at 180 h; moreover, when 2 wt% biochar was combined with 8 wt% reactive MgO expansive additive, the paste achieved ∼80 microstrain expansion (rather than shrinkage) at 180 h, indicating that biochar-supplied water can directly govern the kinetics of both hydration and shrinkage-compensating reactions when expansive phases are present.^50^

By providing a continuous supply of water to the hydrating cement grains, internal curing promotes more complete hydration, reduces self-desiccation stresses, and helps develop a more robust and less cracked microstructure, especially around the biochar particles. This effect is not purely physical; the chemistry of the pore solution and the surface tension effects within the biochar's pore network govern the kinetics of water release, ensuring a sustained supply during the critical early stages of hardening. The combined effects of nucleation and internal curing lead to a denser and stronger interfacial transition zone (ITZ) between the biochar particle and the cement matrix, which is crucial for the overall mechanical performance of the composite. However, a critical comparison of reported outcomes indicates that “internal curing” should not be treated as an unconditional benefit: its effectiveness is contingent on the biochar saturation state, pore connectivity, and surface chemistry, as well as on competing phenomena that can increase capillary stresses or create local defects. For instance, Gupta and Kashani observed that peanut-shell biochar increased the degree of hydration (reported as 13–23%) and improved early-age strength (up to 18–22% at 7 days), yet shrinkage increased with biochar dosage, which they linked to impurities (for example salts) in unwashed feedstock-derived biochar that can perturb early-age transport and hydration chemistry.^51^ Taken together, these case studies suggest that the same porous reservoir that enables internal curing can either refine or compromise the interfacial microstructure depending on whether (i) released water is synchronized with the dominant shrinkage-driving reactions (self-desiccation in low water-to-cement ratio systems), (ii) biochar addition remains below a defect-forming threshold (noted in UHPC when exceeding 1% replacement), and (iii) the biochar surface and ash fraction do not introduce ionic effects that shift shrinkage behavior in the opposite direction.

An important boundary condition is that the internal-curing benefit of biochar is not expected to be equally strong across all binder systems. In low water-to-binder formulations, especially systems prone to self-desiccation, pre-saturated or water-accessible biochar can meaningfully mitigate autogenous shrinkage; in more conventional mixtures with higher available capillary water, the same particles may contribute more through filler, nucleation, and transport-path modification than through a dominant shrinkage-mitigation mechanism.^52,53^

When biochar is co-used with supplementary cementitious materials (SCMs), the governing chemistry is no longer limited to ‘nucleation *vs.* dilution’; it becomes a phase-assemblage problem controlled by pore-solution alkalinity, sulfate balance, carbonate availability, and reactive Al supply. In slag- and fly-ash-blended binders, SCM dissolution consumes Ca(OH)_2_ and drives formation of Al-bearing C–S–H (often denoted C–A–S–H) alongside aluminate hydrates and AFm/LDH-type phases, changing both transport-controlling microstructure and the identity of anion-binding solids. Thermodynamic/experimental studies of SCM systems show that AFm populations can become substantial in slag blends and that carbonate availability can redirect alumina into carboaluminate AFm phases while stabilizing ettringite, illustrating why ‘SCM chemistry’ must be discussed through hydrate assemblage rather than only strength/porosity trends.^54^

Within this SCM context, biochar can influence reactions through three coupled pathways. First, its internal-curing reservoir can sustain later-age hydration and SCM reaction (especially in low w/b), which is relevant for slow-reacting SCMs (*e.g.*, class F fly ash) because water availability and alkalinity govern dissolution kinetics. Second, biochar ash can act as an additional alkali/Si/Al source depending on feedstock and pyrolysis conditions, shifting pore-solution composition and thereby the stability fields of AFt/AFm and the Al-uptake of C–(A)–S–H. Third, biochar-associated carbonate (from mineral ash and/or accelerated carbonation enabled by its pore network) can increase the thermodynamic driving force for forming carboaluminate AFm phases in alumina-bearing blends (notably LC^3^-type systems), which has direct implications for chloride binding because carbonate-AFm and chloride-AFm compete through anion exchange and phase conversion. These cement-chemistry constraints explain why the same ‘biochar addition’ can lead to different hydration/setting outcomes in different SCM systems and why mechanistic interpretation should report binder chemistry (Al_2_O_3_, SO_3_, CO_3_^2−^ sources) alongside biochar properties.^55^

## 3. Pore structure refinement

The durability and mechanical properties of cementitious materials are intrinsically linked to their pore structure—specifically, the total porosity, pore size distribution, and connectivity (tortuosity) of the pore network. One of the most consistently reported benefits of incorporating biochar at optimal dosages is the refinement of this pore structure.^56^ This refinement is a direct consequence of the interfacial chemical and physical mechanisms discussed previously, manifesting as a densification of the matrix. Biochar's influence on the pore structure can be understood through two primary, synergistic mechanisms: the physical filler effect and the chemical nucleation effect.

**Fig. 4:**
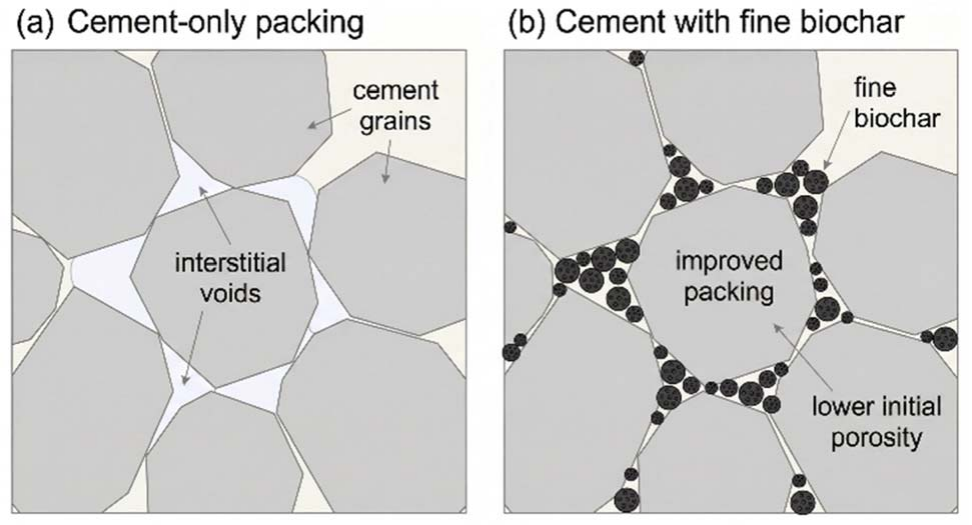
Simplified schematic of the physical filler effect of fine biochar particles. (a) Cement grains packed without fine biochar, leaving relatively large interstitial voids. (b) Fine biochar particles partially occupy the spaces between cement grains, improving particle packing and lowering the initial porosity.

The physical filler effect is most pronounced when using finely ground biochar particles. These particles, being smaller than the unhydrated cement grains, can physically occupy the interstitial spaces between them, as illustrated in Fig. 4. This process, analogous to the effect of other fine fillers like silica fume or limestone powder, improves the packing density of the solid constituents in the fresh paste. Consequently, the initial water-filled volume that will later become the capillary pore network is reduced from the outset. As hydration proceeds, the hydration products grow and fill the remaining spaces. With better initial particle packing, there is less volume to fill, leading to a lower overall porosity in the hardened material^57^ This effect is particularly important in reducing the volume of large capillary pores (typically > 50 nm), which are known to be most detrimental to both mechanical strength and resistance to fluid transport.

Two recent datasets illustrate both the promise and the limits of this “refinement” narrative when it is interrogated with quantitative pore metrics rather than inferred solely from strength and sorptivity trends. First, Lorenzoni *et al.*^58^ examined wood-biochar–cement pastes using a deliberately multi-technique framework, showing why “total porosity” and “transport-relevant pore connectivity” can diverge in biochar systems. Across techniques, they observed that increasing biochar replacement tended to increase measured porosity because the biochar itself is highly porous; however, their micro X-ray computed tomography segmentation simultaneously indicated a pronounced reduction in the volume of large pores in the composite, with the “large pore” fraction reduced by 55.6% at 5 vol% replacement and by 61.1% at 25 vol% replacement relative to the reference paste.^59^ This juxtaposition is mechanistically consistent with a filler-driven packing improvement and/or altered air-void escape through biochar channels,^60^ yet it also highlights a critical interpretive constraint: porosity introduced inside biochar may inflate bulk porosity estimates without proportionally worsening ionic transport, meaning that durability cannot be inferred from porosity magnitude alone without addressing percolation and tortuosity explicitly.^61^

Second, Wu *et al.*^25^ provide a dosage-dependent counterpoint in an ultra-high performance concrete mortar matrix, where mercury intrusion porosimetry captured a modest porosity decrease at 1% biochar replacement but a clear degradation at higher substitutions. Specifically, they report that the 1% biochar mixture exhibited a slight reduction in overall porosity and a downward shift in the cumulative pore volume curve *versus* the control, consistent with micro-filler packing and added nucleation sites;^25^ by contrast, at elevated substitutions the pore structure coarsened and porosity increased substantially, with the authors reporting porosity increases on the order of ∼23.6% and ∼38.5% for 8% and 24% replacement, respectively.^62^ From a critical standpoint, this pattern reinforces that the “physical filler effect” is conditional: once biochar content exceeds the regime where particles primarily occupy interstitial voids, the intrinsic porous volume of biochar, its tendency to agglomerate, and the effective water-demand/workability penalty can generate additional entrapped voids and connected capillary pathways that overwhelm any packing benefit, shifting the system from refinement to net pore-network proliferation.^63^ This is particularly important for the present review's broader durability themes: higher biochar dosages may increase carbon storage potential, but if they concurrently create more connected transport pathways, the expected gains in chloride resistance may not materialize unless immobilization mechanisms compensate for the more permissive pore network.^64^

**Fig. 5:**
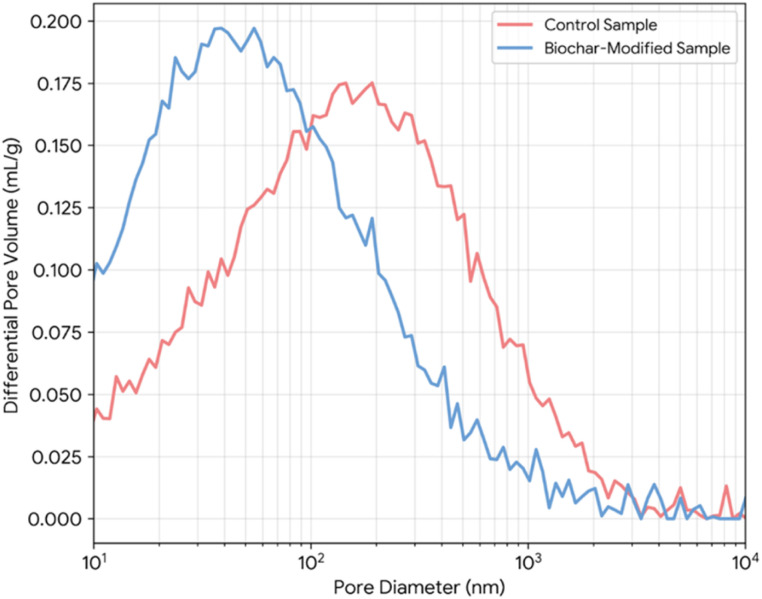
Representative MIP results showing the effect of biochar addition on the pore size distribution of cement paste. Data from our own lab.

The chemical nucleation effect, however, provides a more profound and transformative refinement of the pore structure. As established, biochar surfaces act as preferential sites for the precipitation of C–S–H. This leads to a more disseminated and homogeneous distribution of the hydration products throughout the matrix. Instead of forming thick, dense shells of C–S–H primarily around the large cement grains (leaving larger inter-grain regions to form capillary pores), the C–S–H grows on the countless biochar surfaces dispersed throughout the paste. This “decentralized” growth strategy effectively fills the smaller capillary pores more efficiently, breaking down large, interconnected pore pathways into smaller, more isolated, and tortuous ones.^65^ Experimental evidence from mercury intrusion porosimetry (MIP) consistently demonstrates this phenomenon. For instance, Choi *et al.*^66^ showed that the addition of 1% wood-derived biochar shifted the critical pore diameter to a finer range and significantly reduced the volume of pores larger than 100 nm. This shift towards a higher proportion of harmless or less harmful micropores (<50 nm) is a hallmark of pore structure refinement and is directly correlated with improvements in compressive strength and reductions in permeability. Fig. 5 presents typical MIP results showing this shift in pore size distribution.

**Fig. 6:**
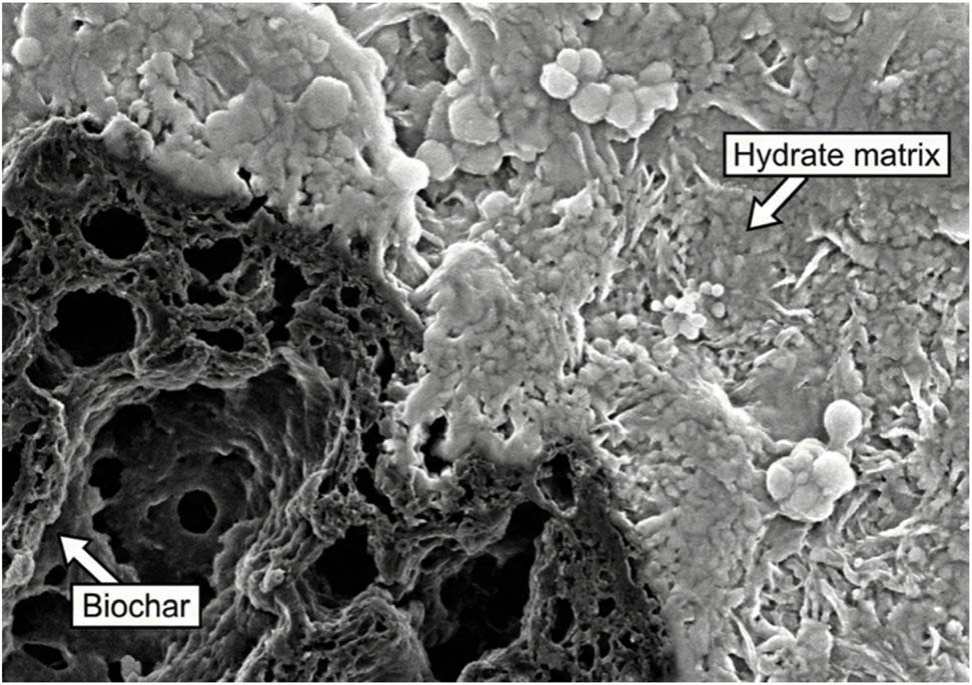
SEM image of the ITZ between a biochar particle (darker, porous region) and the cement hydrate matrix (lighter region). The interface appears dense and well-bonded, with hydration products growing directly on and into the biochar surface. Figure from our own lab.

The combination of these effects leads to a significant densification of the interfacial transition zone (ITZ). In conventional concrete, the ITZ around aggregates is often the weakest link, characterized by higher porosity and larger, more oriented crystals of calcium hydroxide (CH) due to the “wall effect.” When biochar is introduced, its fine particles populate this zone, and its active surface promotes the growth of dense C–S–H, consuming the otherwise weak and porous CH through a potential pozzolanic reaction over the long term. This results in a stronger, less porous ITZ, improving the bond between the paste and aggregates and enhancing the overall mechanical performance of the composite. Scanning electron microscopy (SEM) images, such as the one in Fig. 6, often reveal a seamless and dense interface between biochar particles and the surrounding hydrate matrix, in stark contrast to the porous ITZ typically seen around inert fillers.^67^

However, the effect of biochar on pore structure is highly dependent on its dosage and physical properties, leading to a significant point of contention and a critical optimization challenge. While low dosages (typically 0.5–2% by weight of cement) generally lead to pore refinement, higher dosages can have the opposite effect. A concrete-scale dataset that makes this nonlinearity explicit is provided by Ling *et al.*,^68^ who varied biochar dosage (1–10 wt%) and fineness and found that performance peaks at modest replacement: at 3 wt% biochar, compressive strength increased by up to about 18%, whereas at 10 wt% the 28 day strength fell below the control. Importantly for durability-linked pore connectivity, the same study reported that at 1 wt% biochar the 28 day accelerated carbonation depth decreased by up to 17.9%, and at 1 wt% the 90 day chloride diffusion coefficients clustered around 14–16 × 10^−12^ m^2^ s^−1^ with a measurable optimum at intermediate fineness (14.04 × 10^−12^ m^2^ s^−1^). This case illustrates both the promise and the interpretive tension for pore refinement: carbonation mitigation and reduced chloride diffusivity can coexist at low dosage, but the trend can reverse with increasing dosage because biochar's intrinsic pore volume and the dilution of clinker hydration products begin to dominate transport.

At higher biochar contents, the trade-offs become even clearer and explain why an “optimal dosage” cannot be treated as a universal constant. When the volume fraction of porous particles becomes too large, local nucleation or internal-curing benefits may persist, but connected transport pathways, entrained voids, or weak zones can still grow. This is why pore refinement, chloride resistance, mechanical performance, and freeze-thaw behavior should be interpreted as a multi-objective optimization problem rather than as a single monotonic response to increasing biochar addition.

Pore refinement and ITZ densification must also be evaluated together with carbonation chemistry, because carbon sequestration and durability are mechanistically coupled rather than independent. Carbonation may densify parts of the microstructure and increase measured CO_2_ uptake, but it can simultaneously reduce alkalinity and alter AFm-related chloride binding equilibria. For that reason, chloride durability in biochar systems should be discussed through both transport control and binding stability, especially when carbonation-promoting formulations are proposed as sustainability strategies. Table 2 consolidates representative datasets linking biochar particle characteristics and dosage to porosity-strength trade-offs, underscoring that pore refinement and macrovoid introduction may coexist in the same formulation.

**Table 2:** Influence of biochar particle size and dosage (wt% of cement) on the total porosity and compressive strength of cementitious composites

Biochar particle size	Dosage (wt% cement)	Change in total porosity (%)	Change in compressive strength (%)	Key observation	Reference
<75 µm	1.0	−15.2	+21.5	Significant pore refinement and strength increase	68
<75 µm	5.0	+8.5	−12.0	Increased porosity due to biochar's internal pores and agglomeration	68
150–300 µm	2.0	+4.1	−5.8	Coarse particles increased porosity and weakened the ITZ	49
Nano-biochar (<1 µm)	0.5	−18.0	+28.0	Nano-filler effect provided superior pore refinement	74
As-produced (mixed)	10.0	+35.0	−30.4	High dosage and poor grading dramatically increased porosity	75

## 4. Chloride immobilization mechanisms

The durability of reinforced concrete structures, especially in aggressive environments such as coastal areas or regions where de-icing salts are used, is critically dependent on their ability to resist chloride ion penetration.^69^ Chloride ions can permeate the porous concrete cover and, upon reaching a critical concentration at the surface of the steel reinforcement, depassivate the protective oxide layer and initiate corrosion, which is a primary cause of premature structural degradation.^69^ The resistance to chloride ingress is governed by two factors: the transport properties of the concrete (*i.e.*, its permeability and diffusivity, which are functions of the pore structure) and its chloride binding capacity. Chloride binding refers to the processes by which free chloride ions in the pore solution are immobilized, rendering them unable to contribute to diffusion or corrosion initiation. Biochar modification can influence both of these aspects through a combination of direct and indirect mechanisms.^70^

Chloride binding in conventional cement systems occurs through two main pathways. Chemical binding involves the reaction of chloride ions with the aluminate phases in cement, primarily tricalcium aluminate (C_3_A), to form insoluble calcium chloroaluminate hydrates, most notably Friedel's salt (3CaO·Al_2_O_3_·CaCl_2_·10H_2_O).^71^ Physical binding, or adsorption, is a surface phenomenon where chloride ions are electrostatically or physically adsorbed onto the surfaces of the hydration products, particularly the C–S–H gel, which possesses a high specific surface area and a complex surface charge.^72^

**Fig. 7:**
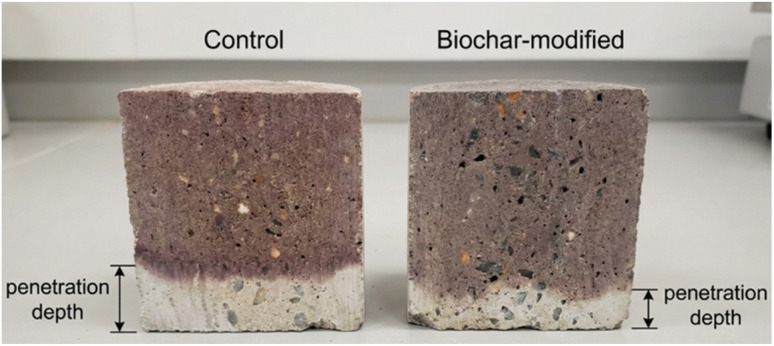
Results from a non-steady-state chloride migration test (NT BUILD 492). The concrete sample is split and sprayed with a silver nitrate solution. Figure from our own lab.

The primary and most widely accepted mechanism by which biochar enhances chloride resistance is indirect, stemming from its profound effect on pore structure refinement. As discussed in the previous section, the nucleation and filler effects of biochar lead to a denser, less porous, and more tortuous matrix. A lower volume of interconnected capillary pores physically restricts the pathways available for chloride diffusion, while increased tortuosity lengthens the effective path an ion must travel to reach the reinforcement.^73,74^ This results in a significantly lower apparent chloride diffusion coefficient, a key parameter in service life prediction models. Numerous studies have confirmed this effect using standard tests like the rapid chloride permeability test (RCPT) and bulk diffusion tests.^75^ For example, Gupta *et al.*^67^ reported a 30% reduction in the charge passed in RCPT for mortar containing 2% rice husk biochar, directly attributing it to the refined pore structure observed *via* MIP. Fig. 7 shows typical results from chloride migration tests, illustrating the reduced penetration depth in biochar-modified samples.

Beyond this well-established physical barrier effect, a more debated and scientifically intriguing question is whether biochar itself can directly contribute to chloride immobilization.^76^ Here, ‘direct immobilization’ refers to chloride partitioning attributable to the biochar phase itself (*e.g.*, adsorption/isotherm uptake in alkaline pore solution), distinct from increased binding by additional AFm or C–S–H phases formed indirectly. The high specific surface area and intricate pore network of biochar suggest a potential for physical adsorption of chloride ions.^77^ Some researchers propose that chloride ions can be adsorbed onto the carbonaceous surfaces within the biochar pores *via* van der Waals forces or other weak physical interactions.^78^ However, direct experimental evidence for this mechanism within the highly alkaline and complex chemical environment of cement pore solution is scarce and often conflicting.^79^ The main argument against significant direct adsorption is the electrostatic repulsion between the negatively charged biochar surface (due to deprotonated functional groups at high pH) and the anionic chloride ions (Cl^−^).^80^

To address this limitation, increasing attention has been given to functionalized or modified biochars rather than untreated carbonaceous particles alone. Conventional oxidative acid or alkali treatments can alter the abundance of oxygen-containing functional groups and surface polarity, whereas amination introduces nitrogen-containing moieties that may change acid–base behavior, cation affinity, and the local interfacial charge environment. In highly alkaline cement pore solution these modifications should not be interpreted as guaranteeing strong direct chloride adsorption, but they can still influence calcium complexation, mineral growth near the biochar surface, and the balance between transport reduction and interfacial immobilization pathways. Table 3 therefore compares chloride-related outcomes by transport test, biochar condition, and inferred mechanism so that physical barrier effects are not conflated with claims of direct chloride immobilization.^81–83^

**Table 3:** Summary of chloride transport performance in biochar-modified cementitious composites as reported in the literature

Biochar type	Dosage (wt% cement)	Test method	Chloride diffusion/migration coefficient (×10^−12^ m^2^ s^−1^)	% reduction *vs.* control	Reference
Wood biochar	1.0	NT BUILD 492	8.5 (*vs.* 12.2 for control)	30.3%	90
Rice husk ash biochar	2.0	Bulk diffusion	4.2 (*vs.* 6.8 for control)	38.2%	69
Sewage sludge biochar	3.0	NT BUILD 492	10.1 (*vs.* 11.5 for control)	12.2%	40
Bamboo biochar	0.5	RCPT (charge passed)	950C (*vs.* 1500C for control)	(N/A, charge passed)	57
Wood waste biochar	5.0	NT BUILD 492	14.5 (*vs.* 12.2 for control)	−18.9% (increase)	91

Another indirect chemical mechanism involves biochar's influence on hydrate assemblage and chloride partitioning, which becomes especially important in SCM-containing binders. By accelerating hydration and increasing the volume and dispersion of C–(A)–S–H, biochar can increase the total surface area available for physical chloride adsorption; however, in slag/fly ash/calcined-clay systems the dominant chemical binding reservoir is typically the AFm family, whose composition (OH^−^/SO_4_^2−^/CO_3_^2−^/Cl^−^ interlayers) is governed by Al availability, sulfate balance, and carbonate activity.^84^ In these binders, any biochar-derived contribution of reactive Si/Al (ash-driven pozzolanicity) or carbonate (mineral ash and/or carbonation promoted by biochar porosity) can shift AFm speciation toward carboaluminates, and these phases can subsequently participate in anion exchange and/or conversion pathways relevant to Friedel's salt formation under chloride exposure.^85^ Cement-chemistry studies on SCM/limestone/metakaolin systems demonstrate that carbonate reacts with alumina to form additional AFm carboaluminate phases and can stabilize ettringite, highlighting that carbonate availability is a first-order control on aluminate hydrate populations; chloride-binding reviews similarly show that SCMs can alter binding capacity in binder-specific ways *via* changes in C–S–H chemistry and AFm abundance.^86^ Accordingly, in biochar–SCM blends, improvements in chloride resistance should be interpreted as a coupled outcome of (i) transport changes (tortuosity/critical pore diameter) and (ii) phase-assemblage changes (AFm identity/amount and its competition between CO_3_^2−^ and Cl^−^), rather than attributing effects to ‘biochar adsorption’ alone.^87^

## 5. Carbon sequestration and long-term stability

The primary environmental driver for incorporating biochar into concrete is its potential for carbon sequestration. This potential is realized through a dual mechanism that combines passive, long-term storage with the potential for active, chemically-driven capture of atmospheric CO_2_, making biochar a compelling tool for developing carbon-negative construction materials.^88^ However, the long-term stability and potential side effects of these sequestration mechanisms, particularly in steel-reinforced concrete, are subjects of intense scholarly investigation and debate.^89^

**Fig. 8:**
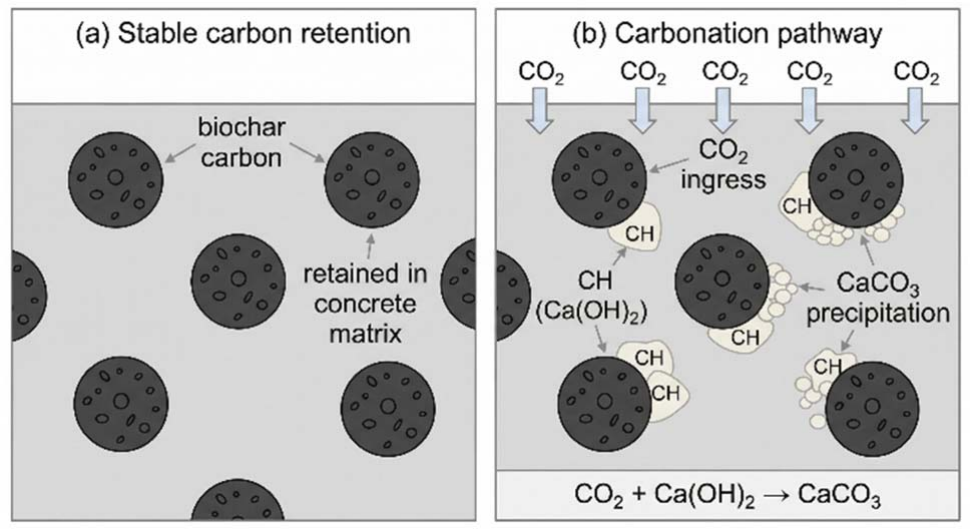
Simplified schematic of the dual carbon sequestration roles of biochar in concrete. (a) Stable biochar carbon is retained within the hardened concrete matrix. (b) Biochar may contribute to local carbonation by influencing CO_2_ ingress and promoting the reaction of CO_2_ with CH (Ca(OH)_2_) to form CaCO_3_ in the surrounding matrix.

The first and most direct mechanism is passive sequestration, also referred to as carbon storage or abatement. The carbon in biomass is part of the fast biogenic carbon cycle. Through photosynthesis, plants capture atmospheric CO_2_, which is then released back into the atmosphere upon decomposition.^90^ The pyrolysis process transforms this biogenic carbon into a highly aromatic and recalcitrant form that is extremely resistant to microbial and chemical degradation.^6^ By incorporating this stable biochar into a concrete matrix, the carbon is effectively sequestered and removed from the atmospheric cycle for the entire service life of the structure, and likely far beyond, even after demolition if the concrete is recycled as aggregate or landfilled. This is illustrated in the schematic in Fig. 8. The amount of carbon sequestered is directly proportional to the amount of biochar added. Given that biochar can have a carbon content exceeding 70–80%, even a small dosage by weight of cement can translate into a significant quantity of sequestered carbon over the vast volumes of concrete produced globally.^91^ For example, replacing 2% of cement with biochar (80% C content) in a cubic meter of concrete (assuming ∼300–350 kg cement per m^3^ concrete and 2 wt% replacement by cement mass) could sequester approximately 5–6 kg of carbon, which translates to 18–22 kg of CO_2_ equivalent.^67^

The second mechanism is the enhancement of active sequestration through accelerated carbonation. Concrete naturally absorbs CO_2_ from the atmosphere in a slow process called carbonation, where CO_2_ reacts with calcium hydroxide (Ca(OH)_2_) to form calcium carbonate (CaCO_3_), a thermodynamically stable mineral. This reaction, Ca(OH)_2_ + CO_2_ → CaCO_3_ + H_2_O, also sequesters carbon. Several studies suggest that the porous network within biochar particles can act as preferential pathways for the ingress of atmospheric CO_2_ deep into the concrete matrix, thereby increasing the rate and depth of carbonation.^92^ The high surface area of biochar may also provide nucleation sites for CaCO_3_ precipitation. This accelerated carbonation not only sequesters additional CO_2_ but can also contribute to matrix densification and strength gain, a process known as carbon curing.^93^Table 4 presents findings from studies investigating the carbon sequestration potential, highlighting the contributions from both passive storage and enhanced carbonation.

**Table 4:** Reported carbon sequestration and carbon-abatement performance of biochar-modified cementitious composites, capturing both (i) passive carbon storage attributable to the biogenic carbon retained in biochar and (ii) active CO_2_ uptake *via* enhanced/accelerated carbonation

Study focus	Biochar dosage (% of cement, unless noted)	Measurement/calculation method	Key quantitative finding
Carbon abatement potential (LCA; biochar + SCM synergy)	Biochar up to 5 wt%; also evaluates 5 wt% biochar + 35 wt% fly ash	Cradle-to-cradle life cycle assessment (Ecoinvent 3.9; LCA software)	5 wt% biochar + 35 wt% fly ash reduced GWP by 23%; “optimal performance” observed at 2 wt% biochar balancing performance and carbon storage
CO_2_ uptake enhancement quantified by TGA (biochar alone *vs.* biochar + FA)	1 wt% biochar; 1 wt% biochar + 10% class C fly ash	Thermogravimetric analysis (TGA) for CO_2_ uptake	Relative to OPC: +42% CO_2_ uptake with 1% biochar; +92% CO_2_ capture capacity with biochar + 10% class C fly ash
Mechanical response under accelerated carbonation (carbonation curing context)	(Same study context as above)	Accelerated carbonation testing + mechanical indices	Under accelerated carbonation: biochar-enriched mortars show 20% higher modulus of elasticity and up to 64% higher toughness indices *vs.* reference carbonated OPC
Carbonation curing + biochar (internal CO_2_ uptake; durability/footprint co-benefits)	1–5% (corn-straw biochar)	Carbonation curing study + environmental evaluation	Biochar addition promoted internal CO_2_ uptake and (per authors) reduced carbon footprint and energy consumption of concrete products (study reports mechanism + directionality; detailed uptake numbers are in full text)
High-volume biochar replacement coupled with carbonation curing (mortar)	10–40% by volume (rice husk biochar); “optimum” noted at 20%	Carbonation curing; CO_2_ uptake quantified by authors	CO_2_ uptake improved up to 46.2% (*vs.* control). “Saturated RHB-added mortar” showed highest CO_2_ uptake of 17.1%, higher than unsaturated RHB-added mortar

**Fig. 9:**
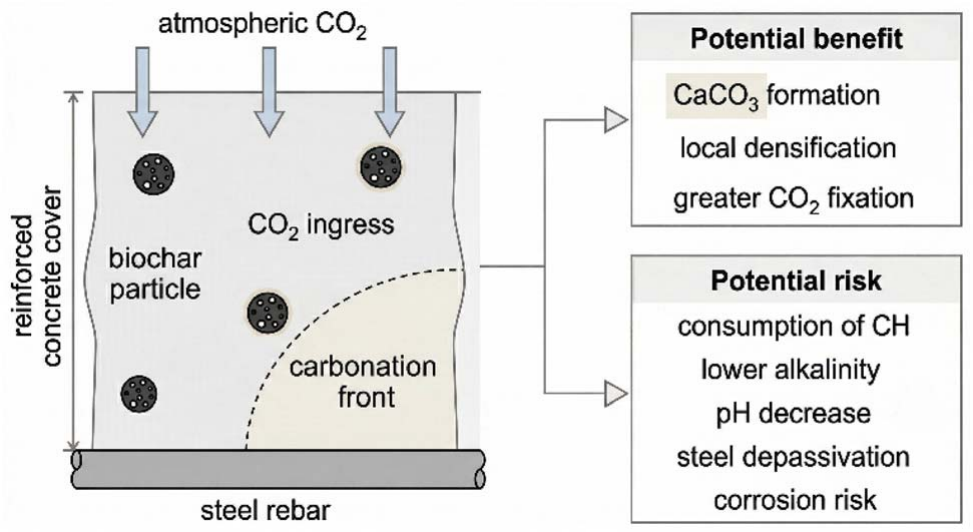
The carbonation–corrosion dilemma in biochar-modified reinforced concrete. While accelerated carbonation (top path) enhances carbon sequestration and densifies the matrix, it also lowers the pH. If the carbonation front reaches the steel rebar, it can initiate corrosion, compromising structural integrity (bottom path). This highlights the critical need for careful design and long-term monitoring.

Despite these promising benefits, the long-term stability and consequences of biochar-induced carbon sequestration are a major point of controversy. The central issue revolves around the integrity of steel reinforcement. The high alkalinity of concrete pore solution (pH > 12.5) creates a passive film on the surface of embedded steel, protecting it from corrosion. The carbonation reaction consumes Ca(OH)_2_, the primary buffer responsible for this high pH. If the carbonation front advances to the depth of the rebar, the pH can drop to around 9, destroying the passive layer and leaving the steel vulnerable to corrosion in the presence of oxygen and moisture. Therefore, by potentially accelerating carbonation, biochar could inadvertently reduce the service life of reinforced concrete structures. This is the core of the scholarly debate: a mechanism that is beneficial for carbon sequestration in unreinforced concrete could be highly detrimental in reinforced applications, which constitute the vast majority of modern construction. Fig. 9 illustrates this critical trade-off.

The carbonation-related corrosion risk should also be interpreted as a chloride-binding problem in addition to a pH problem. Carbonation can destabilize AFm phases that previously immobilized chlorides, thereby increasing the free chloride fraction at the same time that alkalinity decreases. In other words, active CO_2_ sequestration may intensify corrosion risk from two fronts simultaneously: by weakening steel passivation and by remobilizing chlorides that were formerly bound within the hydrate assemblage.^94–96^

The long-term stability of the sequestered carbon within the biochar itself is generally considered to be very high. Protected from UV radiation and microbial activity within the inert, alkaline concrete matrix, the half-life of biochar carbon is estimated to be in the order of centuries to millennia.^97^ The key uncertainty is not whether the carbon in biochar is stable, but rather how the biochar's presence affects the long-term durability of the entire composite system. There is a profound lack of long-term field data. Most studies are laboratory-based and of short duration (days to months). There are virtually no documented field trials assessing the performance of biochar-amended reinforced concrete over decades. This research gap is a significant barrier to its widespread adoption in critical infrastructure. Proponents argue that the pore refinement caused by biochar can create a matrix so impermeable that the rate of CO_2_ ingress, despite the internal pathways, is still slower than in lower-quality conventional concrete, thus offsetting the risk. Opponents argue that the risk of accelerated carbonation is too great to ignore without extensive, multi-decade validation. Resolving this debate is arguably the most critical challenge facing the field. Table 5 lists common experimental techniques used to characterize the biochar–cement interface and the information they provide. This array of techniques allows researchers to piece together a comprehensive picture of the complex interactions, from hydration kinetics to microstructural evolution and phase composition.

**Table 5:** Common experimental techniques used to interrogate the biochar–cement interface and associated microstructural evolution

Experimental technique	Information obtained about biochar–cement interface	Strengths	Limitations
Scanning electron microscopy (SEM)/energy-dispersive X-ray spectroscopy (EDS)	Microstructure, morphology of ITZ, spatial distribution of hydration products, elemental mapping of the interface	High-resolution imaging, direct visualization of the interface, qualitative chemical analysis	2D imaging of a 3D structure, requires high vacuum, potential for sample preparation artifacts
Isothermal calorimetry	Heat flow and cumulative heat release during hydration	Quantitative data on hydration kinetics (acceleration/delay), effect on C–S–H formation rate	Provides bulk information, does not directly probe the interface
Thermogravimetric analysis (TGA)	Quantification of hydration products (CH, CaCO_3_), degree of hydration, bound water content	Quantitative analysis of phase composition, can track pozzolanic reaction and carbonation	Decomposition temperatures of some phases can overlap
Mercury intrusion porosimetry (MIP)	Total porosity, pore size distribution, critical pore diameter	Provides quantitative data on the pore structure refinement	Uses high pressure which can damage the pore structure, does not measure disconnected pores
Fourier-transform infrared spectroscopy (FTIR)/X-ray diffraction (XRD)	Identification of chemical functional groups and crystalline phases (CH, ettringite, CaCO_3_)	Provides chemical and mineralogical information about the reaction products	Primarily qualitative or semi-quantitative, provides bulk sample information

## 6. Conclusions and future perspectives

The incorporation of biochar into cementitious materials represents a compelling, multifaceted strategy for enhancing sustainability in the construction industry. The interfacial chemistry between biochar and the hydrating cement matrix is the linchpin that governs the material's ultimate performance, from its mechanical properties to its long-term durability and carbon footprint. This review has critically examined the state-of-the-art understanding of these interactions, focusing on three pivotal outcomes: pore structure refinement, chloride immobilization, and carbon sequestration.

The evidence is strong that biochar acts as a potent microstructural engineering agent. Through a synergistic combination of physical filling and chemically-driven nucleation, optimized additions of biochar lead to a demonstrably refined pore structure. This results in a denser, less permeable matrix with a higher proportion of finer, less harmful pores, which is the root cause of observed improvements in compressive strength and resistance to fluid ingress. However, this effect is highly sensitive to biochar properties and dosage, with excessive amounts proving detrimental by introducing porosity and creating weak zones.

The role of biochar in enhancing chloride resistance is primarily an indirect consequence of this microstructural refinement. The densified and more tortuous pore network presents a formidable physical barrier to chloride diffusion. The debate over biochar's capacity for direct chloride binding remains largely unresolved, with current evidence suggesting its contribution is minor compared to the physical barrier effect and its indirect enhancement of C–S–H formation. This area, particularly concerning the use of functionalized biochars, warrants significant further investigation.

As a carbon sequestration agent, biochar offers a powerful dual mechanism of passive storage of recalcitrant carbon and potential acceleration of active CO_2_ capture through carbonation. While the stability of the sequestered carbon within the biochar itself is high, the accelerated carbonation poses a critical and unresolved risk to the durability of reinforced concrete by potentially promoting steel corrosion. This trade-off between enhanced carbon sequestration and structural integrity is the most significant controversy and barrier to the widespread application of biochar in modern infrastructure. Based on this comprehensive analysis, several critical knowledge gaps and future research directions can be identified:

(1) Advanced interfacial characterization: there is a need to move beyond bulk characterization methods and employ advanced, *in situ* techniques such as nanoindentation, atomic force microscopy (AFM), and synchrotron-based X-ray microscopy to directly probe the mechanical properties and chemical composition of the biochar–hydrate interface at the nanoscale. Molecular dynamics simulations could further elucidate the fundamental binding energies and reaction pathways.

(2) Biochar engineering and functionalization: future research should focus on tailoring biochar properties for specific functions. This includes optimizing pyrolysis conditions to control surface chemistry and porosity, and developing novel functionalization methods to enhance, for example, chloride binding capacity or to selectively promote beneficial reactions while mitigating detrimental ones.

(3) Long-term durability and field trials: the most pressing need is for comprehensive, multi-decade field studies on biochar-modified reinforced concrete structures under realistic environmental conditions. Long-term monitoring of carbonation depth, corrosion initiation, and mechanical performance is essential to validate laboratory findings and develop reliable service life prediction models.

While long-term field exposure remains essential, short-term proxy frameworks can still be made more informative by combining accelerated carbonation, chloride migration or diffusion testing before and after pre-carbonation, cyclic wetting-drying chloride exposure, and electrochemical corrosion monitoring under coupled deterioration conditions. These experiments should be paired with thermodynamic, reactive-transport, and pore-structure-aware predictive modeling so that accelerated observations can be translated into mechanistically grounded service-life interpretation.^98,99^

(4) Standardization and life cycle assessment: for biochar to transition from a laboratory curiosity to a mainstream construction material, standardized specifications for its physical and chemical properties are required. Concurrently, rigorous and holistic life cycle assessments (LCAs) are necessary to quantify the net environmental benefits, considering the entire value chain from biomass sourcing and pyrolysis to the end-of-life of the structure.

In conclusion, the interfacial chemistry of biochar-modified cementitious materials is a rich and complex field that holds the key to unlocking a new generation of sustainable and durable construction materials. While significant progress has been made in understanding the fundamental mechanisms, overcoming the challenges related to optimization, long-term performance, and potential negative side-effects will require a concerted and interdisciplinary research effort. If these challenges can be met, biochar stands to play a pivotal role in decarbonizing the built environment.

## Author contributions

L. W. contributed to conceptualization, methodology, literature investigation, and data curation. L. W. also performed formal analysis, prepared the figures and visualizations, and wrote the original draft of the manuscript. L. S. contributed to conceptualization, formal analysis, and critical interpretation of the literature, and was responsible for writing – review and editing. L. S. provided supervision, project administration, and funding acquisition. All authors have read and agreed to the published version of the manuscript.

## Conflicts of interest

There are no conflicts to declare.

## Data Availability

No primary research results, software or code have been included and no new data were generated or analysed as part of this review.
